# Tennessee hospital noncompliance with price transparency legislation for 8 common laboratory tests

**DOI:** 10.1093/ajcp/aqae057

**Published:** 2024-05-11

**Authors:** Stephanie A Hart, Ayesha Khan, Garrett S Booth, Joesph R Wiencek

**Affiliations:** Department of Pathology, Microbiology and Immunology, Vanderbilt University School of Medicine, Nashville, TN, US; Department of Pathology, Microbiology and Immunology, Vanderbilt University School of Medicine, Nashville, TN, US; Department of Pathology, Microbiology and Immunology, Vanderbilt University School of Medicine, Nashville, TN, US; Department of Pathology, Microbiology and Immunology, Vanderbilt University School of Medicine, Nashville, TN, US

**Keywords:** price transparency, compliance, CMS final rule, laboratory tests

## Abstract

**Objectives:**

The goal of this study was to assess hospital compliance with federal price transparency mandates and barriers to pricing information in Tennessee.

**Methods:**

All hospitals websites were queried for gross, cash, and BlueCross BlueShield of Tennessee prices for 8 high-frequency laboratory tests in 2 Centers for Medicare & Medicaid Services–mandated pricing sources: (1) a machine-readable file of all available services and (2) a consumer-friendly display of 300 shoppable services. Barriers, including click counts, data availability, and intrahospital price discrepancies, were noted.

**Results:**

Of the 145 Tennessee hospitals assessed, 97.2% were noncompliant with the Centers for Medicare & Medicaid Services final rule. Subanalysis of available machine-readable files, price estimators, and shoppable services files demonstrated 49.6%, 95.1%, and 78.6% noncompliance, respectively. Barriers to pricing information included requiring protected health information (55.9%), missing at least 1 pricing source (7.6%), having no pricing sources available (6.2%), and involving more than 3 clicks to access the cash price in machine-readable files (54.1%) and price estimators (68.6%.) Average intrahospital discrepancy for basic metabolic panel cash prices across pricing sources was $101.30 (range, $0-1012.40).

**Conclusions:**

Our study showed high levels of noncompliance with price transparency laws, inconsistent and inaccessible pricing, and continued challenges facing patients in Tennessee.

**Key Points**

- Federal price transparency law requires hospitals to provide 5 price points within 2 pricing sources (a machine-readable file and a user-friendly display of shoppable services).
- Barriers to accessing pricing information included high click counts, intrahospital discrepancies across and missing information in pricing sources, and protected health information disclosure requirements.
- High rates of noncompliance with the federal price transparency law continue to be a barrier for patients trying to find affordable health care.

## INTRODUCTION

Price transparency is a proposed mechanism to combat rising health care costs. With approximately 28.1 million uninsured patients in the United States and 43.4% of insured patients enrolled in high-deductible health care plans, a substantial proportion of patients are responsible for the full cost of their health care spending.^1,2^ Patients with access to accurate health care cost information are more likely to make informed decisions and better understand their out-of-pocket costs.^3,4^ Similarly, price transparency can increase market competition among insurers, potentially leading to a decrease in health care costs and variability.^3,5-7^

On January 1, 2021, the Centers for Medicare & Medicaid Services (CMS) enacted the Hospital Price Transparency final rule (final rule). The final rule mandates that health care institutions provide 2 pricing sources: (1) a machine-readable file of all available services and (2) a consumer-friendly display of 300 shoppable services (70 specified by CMS and 230 selected by the hospital). The consumer-friendly display can be in the format of a shoppable services file or a price estimator. In addition, both mandated pricing sources are required to include 5 price points: gross price, discounted cash price, payer-specific negotiated charges, and deidentified minimum and maximum negotiated charges for each service.^8^ These cost sources must be prominently displayed on the hospital website, easily accessible with the fewest clicks, and available without barriers such as paying a fee or inputting protected health information to access the information.^8^ Full implementation of price transparency across the United States has been estimated to save between $17.6 billion and $80.7 billion by 2025; however, it is unclear whether patients, insurance companies, and patient employers would benefit from these savings.^4,6^

Health care institutions’ noncompliance with the federal law can lead to a range of penalties, including a warning notice, a request for a corrective action plan, and ultimately fines.^8^ As of January 1, 2022, CMS has increased the fine to $300 per day for hospitals with 30 or fewer beds and up to $5500 per day for hospitals with more than 30 beds.^9,10^ Despite the federal regulations and notable penalties, however, price transparency remains inaccessible.^5,6,11-16^

By defining adherence as having all 5 price points within hospital machine-readable files and user-friendly displays of shoppable services, the study by Haque et al^13^ was the first to demonstrate low compliance 9 months following the enactment of the final rule across all hospitals in the United States. Several follow-up investigations showed continued low compliance, but these studies focused on mandated price points within a machine-readable file without properly addressing pricing availability in a user-friendly display.^5,11-13,17-19^ Variations in compliance were also seen across and within regions of a particular state, with no consensus on any particular factor.^13,17^ Jiang et al^17^ found that compliance was strongly associated with peer hospital compliance within a region, information technology preparedness, for-profit hospitals, system-affiliated hospitals, large hospital size, nonurban hospitals, concentrated hospital markets, and more financial resources. Conversely, a separate study found that compliance was positively associated with lower revenue per patient per day, unconcentrated hospital markets, and urban areas.^13^ At the state level, Triana et al^12^ estimated that 16% of the 88 short-term acute-care hospitals in Tennessee were compliant based on machine-readable files alone.

Our study is the first to reevaluate all licensed hospitals within Tennessee, reexplore compliance with the law for both required pricing sources, attempt to validate publicly available pricing data through hospital finance departments, and assess continued compliance challenges through the lens of 8 common laboratory tests 19 months after final rule implementation.

## METHODS

### Study Design and Overview

This study was performed using publicly available online data. Therefore, an institutional board review was not required. To assess hospital compliance and barriers to pricing information within Tennessee, we assessed all licensed health care facilities as of May 3, 2023, as determined using the Tennessee Department of Health Board for Licensing Health Care Facilities.^20^ One hospital was excluded because it closed before data collection began.

In August 2023, we queried all Tennessee hospital websites for the availability of both pricing sources: machine-readable files and user-friendly displays of shoppable services (shoppable services files or price estimator). Three price points (gross, discounted cash, and BlueCross BlueShield of Tennessee) for 8 high-frequency laboratory tests with associated *Current Procedural Terminology (CPT)* identifiers were assessed.^21^ The laboratory tests included basic metabolic panel (BMP) (*CPT* 80048); complete blood cell count (CBC) without differential (*CPT* 85027); CBC automated with differential (*CPT* 85025); vitamin D (*CPT* 82306); hemoglobin, glycated (*CPT* HbA_1c_) (83036); infectious agent detection by nucleic acid, *Chlamydia* (*CPT* 87491); automated urinalysis (*CPT* 81001); and nonautomated urinalysis (*CPT* 81000). A standardized form letter was also sent to the hospital finance department at each institution to request gross, discounted cash, and BlueCross BlueShield of Tennessee prices. Hospitals were given 30 days to respond.

### Compliance

Compliance of a pricing source (ie, the machine-readable file or user-friendly display of shoppable services) was defined as the hospital having the discounted cash and BlueCross BlueShield of Tennessee price available for BMP (*CPT* 80048), urinalysis (*CPT* 81001 or 81000), and CBC (*CPT* 85025 or 85027) in the pricing source. These *CPT* laboratory test identifiers were selected as a subset of the 70 CMS-specified shoppable services the final rule mandated.^22^ Overall compliance was defined as a hospital having both a compliant machine-readable file and user-friendly display of shoppable services, in accordance with federal law.^8^ Price availability as well as the compliance of the machine-readable file and user-friendly display of shoppable services were recorded. A hospital was considered noncompliant if 1 or more of the mandated pricing sources or 1 or more of the prices within the available pricing sources were missing.

### Barriers to Pricing Information

The number of clicks from the hospital home page to pricing information, the amount of protected health information required to access pricing information, and intrahospital price discrepancies were recorded. When more than 1 pricing source was available at an individual hospital or a hospital representative responded to our form letter, we used BMP cash price as a proxy to determine whether there was a discrepancy between the available prices. Intrahospital price discrepancy was calculated as the difference between the maximum and minimum cash prices available for BMP at a given institution. A click count greater than 3 was noted to be a barrier to pricing information.^11^

### Statistical Analysis

Statistics analysis was performed using Microsoft Excel, version 16.16.27, and Stata, version 18, statistical software (StataCorp). Totals, averages, and ranges were calculated.

## RESULTS

### Compliance

Of the 145 Tennessee hospitals included in the study, the noncompliance rate with the final rule was 97.2% (141/145). In accordance with the final rule, a compliant hospital must have both mandated pricing sources available, and both sources must be compliant. Subanalyses showing pricing source availability and noncompliance demonstrated that machine-readable files, price estimators, and shoppable services files were available at 93.1% (135/145), 70.3% (102/145), and 19.3% (28/145) of hospitals, respectively. Of the available pricing sources, 49.6% (67/135) of machine-readable files, 95.1% (97/102) of price estimators, and 78.6% (22/28) of shoppable services files were found to be noncompliant. Overall, 86.2% (125/145) of hospitals had both mandated pricing sources available, but 96.8% (121/125) were noncompliant Table 1.

**TABLE 1:** Subanalysis of Noncompliance at Hospitals in Tennessee

Pricing source	Pricing source noncompliant, n/N (%)	Pricing source available, n/N (%)	Cash price unavailable in pricing source, n/N (%)	BlueCross BlueShield of Tennessee price unavailable in pricing source, n/N (%)
Both mandated pricing sources^a^	121/125 (96.8)	125/145 (86.2)	60/125 (48.0)	121/125 (96.8)
Machine-readable file	67/135 (49.6)	135/145 (93.0)	19/135 (14.1)	67/135 (49.6)
Price estimator	97/102 (95.1)	102/145 (70.3)	45/102 (44.1)	97/102 (95.1)
Shoppable services file	22/28 (78.6)^b^	28/145 (19.3)	12/28 (42.9)	21/28 (75.0)

^a^Overall compliance is defined as cash and BlueCross BlueShield of Tennessee price availability within both mandated pricing sources—that is, a machine-readable file and a user-friendly display of shoppable services. The user-friendly display of shoppable services can be in the form of a price estimator or shoppable services file. A hospital is noncompliant if 1 or more of the mandated pricing sources or 1 or more of the prices within the available pricing sources are missing.

^b^One hospital provided the BlueCross BlueShield of Tennessee price but not the cash price in the institution’s shoppable services file.

### Barriers to Pricing Information

In addition to the missing pricing data, 55.9% of hospitals required protected health information to access pricing data in price estimators, 7.6% of hospitals were missing at least 1 pricing source, and 6.2% had no pricing sources available. The median total number of clicks from home page to cash price in the machine-readable file and price estimator was 4 (range, 1-7) and 10.5 (range, 6-15), respectively. The majority of machine-readable files (54.1%) and price estimators (68.6%) involved more than 3 clicks to navigate from the hospital home page to the cash price. The average intrahospital price discrepancy between different pricing sources was $101.30 (range, $0-1012.40). To access cash and BlueCross BlueShield of Tennessee price, protected health information was required in 26.5% and 79.4% of machine-readable files and price estimators, respectively.

## DISCUSSION

Our analysis demonstrates 97.2% overall noncompliance with the final rule for 8 common laboratory tests and multiple barriers to accessing pricing information, including high click counts, intrahospital discrepancy across pricing sources, missing information, and protected health information disclosure requirements. As of November 20, 2023, only 14 US hospitals have received civil monetary penalty notices from CMS, half are actively appealing the penalties, and it is unclear how many of the remaining hospitals have paid the fines.^23,24^ Despite the low level of compliance demonstrated in this study, none of the Tennessee hospitals have received notices, according to the CMS enforcement disclosures, which may contribute to delayed compliance with federal regulations.^23^

Although the final rule has provided some transparency in hospital pricing, the effects of the rule will remain limited by the level of compliance. We described multiple barriers to accessing pricing information, including missing pricing information, high click counts, and intrahospital discrepant pricing. These factors make navigating the health care system a continuous barrier for patients seeking access to affordable services. In particular, when discrepant cash prices are given for the same service in the machine-readable file and the price estimator, patients cannot determine what price they will pay. In many cases, through our correspondence with hospital representatives to clarify pricing, we received different cash price estimates for 1 test from 2 representatives from the same hospital. Furthermore, the estimates the representatives provided did not correspond to any publicly available pricing within the hospitals’ pricing sources. This finding further demonstrates that pricing can be ambiguous and difficult to interpret^16^—specifically, regardless of whether it was accessed using the hospital’s publicly listed website prices or by reaching out directly to the hospital’s finance department for an estimate, health care pricing remains inaccessible, convoluted, and inconsistent.

The overall generalizability of our findings may not be applicable throughout the United States; however, this statewide pricing assessment is the first to evaluate the compliance with the final rule nearly 2 years after its enactment. We defined compliance as cash and BlueCross BlueShield of Tennessee price availability within a machine-readable file and user-friendly display of shoppable charges. This definition covers 2 of the 5 mandated price points and only 1 of many insurers within the state. We selected and analyzed 8 *CPT* laboratory test identifiers out of all 70 CMS-specified shoppable services. Unlike the majority of previous studies that defined compliance based on pricing availability in the machine-readable files alone, however, our definition of compliance was stricter and representative of the final rule requirements. If we had investigated all 5 price points and a larger range of services, is it plausible that overall noncompliance with federal law may have been even higher than the 97.2% we identified.

Additional challenges we identified through this investigation were the lack of standardization in the method of displaying hospital price transparency resources and formatting of files. Navigating to hospital price transparency pages was difficult because links for price transparency were located under different menus on the hospital website home page, including the “patients and visitors,” “about us,” or “financial services” tabs. Standardizing the placement by providing a “price transparency” button directly on the hospital’s home page to access both mandated pricing sources could eliminate some current barriers by reducing the time and number of clicks required to access pricing information. The differences in machine-readable file formats among hospitals create difficulties in comparing files across health care facilities. Hospitals use multiple file types, such as plain text (.txt), JavaScript Object Notation (.json), Excel (.xlsx), comma-separated values (.csv), and PDF files, and there is no unified method of abbreviating insurers and health plans, labelling columns, formatting the files, presenting the information, or distinguishing between inpatient and outpatient prices.^25^

Price estimators are also highly variable because of their search functions and the number of clicks required within the tool. As demonstrated, 55.9% of all Tennessee hospitals have noncompliant price estimators, forcing patients to provide protected health information when (1) no online documentation is available to disclose how this information would be protected and (2) it is not necessary for compliance. In addition, many price estimators do not consider deductibles or out-of-pocket maximums when providing a patient’s true out-of-pocket costs. Price estimators can be improved by removing the protected health information requirement and providing a tool to better estimate a patient’s overall cost responsibility.

There has been renewed bipartisan interest in price transparency in health care. Specifically, the US Congress passed the Lower Costs, More Transparency Act (the Act) in December 2023 to codify hospital price transparency.^26^ As the Act is brought in front of the US Senate, it will be important for studies to address the sustained or complete deficiencies of the current price transparency legislation.

The new Act aims to improve upon some of the challenges we have noted in the current statute outlined in the final rule, including enforcing harsher penalties, standardizing the method of presentation and format of public standard charges, and investigating the utility and availability of health care price transparency tools.^26^ Regarding stricter penalties, the Act proposes increasing fines as the period of noncompliance increases. For example, a hospital with fewer than 30 beds would be fined $300 per day, $400 per day if noncompliance continues for a year, and between $500,000 and $1,000,000 if the hospital receives more than 2 notices of noncompliance during a 1-year period. Larger hospitals (those with more than 500 beds) would be fined $25 per bed per day, $35 per bed per day if noncompliance continues for a year, and between $5,000,000 and $10,000,000 if they received more than 2 noncompliance notices in a single year.^26^ If enforcement of these stricter penalties continues to be as inconsistent as seen with that of the final rule, however, compliance is likely to remain low.^23^

In addition, the Act is not specific regarding the standardization of machine-readable files or user-friendly displays of shoppable services. Through the Act, the secretary of the US Department of Health and Human Services is tasked with creating a uniform method of displaying and formatting pricing information without providing suggestions. The Act also enlists the comptroller general of the United States to evaluate the usefulness, availability, and compliance of health care price transparency tools to recommend ways to make them more efficient, accurate, user friendly, and comparable across institutions.^26^

Future legislation should focus on increasing health care affordability and accessibility as well as providing concrete guidance on standardizing the way hospitals display and provide pricing information to patients. Our study highlights continued shortcomings of our current health care price transparency legislation and challenges patients face in accessing reliable pricing data for common laboratory tests in Tennessee. Lawmakers should consider the lessons from the final rule to ensure compliance so that it is easier for patients to access, understand, and compare pricing information across hospitals and services.
